# Family Planning for People with Multiple Sclerosis in Saudi Arabia: an Expert Consensus

**DOI:** 10.1155/2021/6667006

**Published:** 2021-02-15

**Authors:** Mohammed Al Jumah, Yaser Al Malik, Nuha M. AlKhawajah, Jameelah Saeedi, Ibtisam AlThubaiti, Saeed Bohlega, Reem F. Bunyan, Edward J. Cupler, Ahmed ElBoghdady, Ahmed Hassan, Eman Nassim Ali, Marinella Clerico

**Affiliations:** ^1^Neurology Department, King Fahad Medical City, Riyadh, Saudi Arabia; ^2^College of Medicine, King Saud Bin Abdulaziz University for Health Sciences, Riyadh, Saudi Arabia; ^3^Division of Neurology, King Abdulaziz Medical City (National Guard Health Affairs), Riyadh, Saudi Arabia; ^4^College of Medicine, King Saud University, Riyadh, Saudi Arabia; ^5^Division of Neurology, King Saud University Medical City, Riyadh, Saudi Arabia; ^6^Neurology Department, King Abdullah Bin Abdulaziz University Hospital, Riyadh, Saudi Arabia; ^7^Neurology Department, King Fahad Military Medical Complex Dhahran, Dammam Eastern Region, Saudi Arabia; ^8^Department of Neurosciences, King Faisal Specialist Hospital and Research Center, Riyadh, Saudi Arabia; ^9^Neurology Department, King Fahad Specialist Hospital Dammam, Dammam Eastern Region, Saudi Arabia; ^10^Department of Neurosciences, King Faisal Specialist Hospital and Research Center, Jeddah, Saudi Arabia; ^11^Merck KGaA, Riyadh, Saudi Arabia; ^12^Clinical and Biological Sciences Department, University of Torino, Orbassano, Italy

## Abstract

More than half of all patients with multiple sclerosis (MS) in the Kingdom of Saudi Arabia (KSA) are women of childbearing age. Raising a family is an important life goal for women in our region of the world. However, fears and misconceptions about the clinical course of relapsing-remitting MS (RRMS) and the effects of disease-modifying drugs (DMDs) on the foetus have led many women to reduce their expectations of raising a family, sometimes even to the point of avoiding pregnancy altogether. The increase in the number of DMDs available to manage RRMS and recent studies on their effects in pregnancy have broadened management options for these women. Interferon beta now has an indication in Europe for use during pregnancy (according to clinical need) and can be used during breastfeeding. Glatiramer acetate is a further possible option for women with lower levels of RRMS disease activity who are, or about to become, pregnant; natalizumab may be used up to 30 weeks in patients with higher levels of disease activity. Where possible, physicians need to support and encourage women to pursue their dream of a fulfilling family life, supported where necessary by active interventions for RRMS that are increasingly evidence based.

## 1. Introduction

Relapsing-remitting multiple sclerosis (RRMS) commonly arises before middle age, at a time when families are likely to be planning to have children [1]. Recent (2020) survey data from a registry in the Kingdom of Saudi Arabia (KSA) showed that about three of every four patients with MS were aged 40 years or less at the time of their MS diagnosis [2]. Moreover, two-thirds of Saudi MS patients in this study were females, suggesting that about half of patients with MS in KSA are women of childbearing potential [2]. In addition, the prevalence of MS has been rising in recent decades, suggesting an increasing burden of MS among this population [2].

RRMS per se does not appear to exert an adverse effect on the course or outcome of pregnancy [3]. But, there is evidence, including in women from the Middle East, that the presence of MS leads women to avoid pregnancy (perhaps completely), due to fears or concerns about the effect of MS on their general health, the adverse effects of MS treatments on the pregnancy or on their fertility, and possible limitations of the use of fertility treatments once MS has been diagnosed [4, 5]. Misconceptions about MS and pregnancy were common among a sample of patients in the USA [6]. Women with MS, and their partners, have the same right to pursue family life as anyone else and should be encouraged to do so [1]. However, limitations on the use of most DMDs during pregnancy (discussed below) complicate the management of their MS at this time.

The frequency of RRMS relapses decreases during the second and third trimesters, but this risk does not disappear, on average [7]. In addition, some studies have shown that there is an increased risk of relapses in the months following delivery [7, 8] although this was not found in a US cohort recently [9]. Continued MS disease activity depletes the capacity of the central nervous system to recover from relapses, and there is a consensus that early and continuous medical intervention in people with MS at risk of relapses is likely to ameliorate progression of disability over the long term, in KSA [10], as elsewhere [11].

A diagnosis of RRMS therefore has the potential to disrupt normal family life for many couples, where the need to facilitate a normal family life as possible must be balanced with preserving the mother's long-term outcome. An expert consensus on the management of RRMS during pregnancy is available for the UK [12], but guidance for countries such as KSA is lacking. In this article, we, a group of physicians from KSA with expertise in the management of MS, provide consensus guidance on the management of RRMS during pregnancy, with special reference to the application of DMD-based therapy for these patients.

## 2. Background: Family Planning in Saudi Arabia

Raising a family is an important life goal for people in KSA, and women in that country (who did not have MS) indicated in a survey a desire to have up to five children in some regions [13]. DMDs that have a strict contraindication in pregnancy require the application of continuous contraception, however. Contraception is accepted by many Saudis, mostly oral contraceptives or intrauterine devices, either to prevent a pregnancy or as a way of spacing out births [13, 14]. Survey conducted in KSA found that a majority of women were using contraception, although supported by medical advice to only a limited extent [14, 15]. Women with higher levels of education and older women with larger existing families are more likely to use contraception [14, 16].

Limited access to contraception, fear of side-effects, religious concerns, and opposition from the husband were important barriers to the use of contraception for Saudi women, in a survey published in 2018 [16]. Data on the proportion of unplanned pregnancies in KSA are scarce, although frequencies of 12% [17] and 23% [18] of all pregnancies have been reported for Saudi women. The information summarised above suggests that many, but not all, Saudi women of childbearing age with MS are prepared to use contraception, but a need to do this due to DMD treatment will often conflict with the desire for a large family.

## 3. Disease-Modifying Drugs and Pregnancy

### 3.1. Therapeutic Indications in Europe and in the USA

Recent years have seen a considerable expansion in the number of DMDs available for the management of RRMS. Among currently available indicated for therapeutic use in people with RRMS, alemtuzumab, cladribine tablets, fingolimod, natalizumab, and ocrelizumab are considered high-efficacy DMDs, usually reserved for use in patients with a history of higher disease activity [19]. The labelling of DMDs in Europe (Summary of Product Characteristics) and US (Prescribing Information) does not provide rules for prescribing that are mandatory in KSA, but they do provide useful sources of information on the level of risk to a pregnancy associated with them. This section provides a brief overview of contraindications and cautions to the use of DMDs in these labels.

At the time of writing, only interferon beta (INF*β*) has a clear indication for use during pregnancy, where this is justified by clinical need, according to a recent update of its European label. Other DMDs that are not formally contraindicated in Europe for use during pregnancy are glatiramer acetate (GA), dimethyl fumarate (DMF), alemtuzumab, natalizumab, and ocrelizumab: the European labels for all of these DMDs carry a statement to the effect that they should be avoided during pregnancy and used only when their benefit clearly outweighs the risks to the pregnancy. Teriflunomide, cladribine tablets, fingolimod, and siponimod are formally contraindicated during pregnancy. Labelling from the USA is more restrictive. The absolute contraindications for fingolimod, siponimod, cladribine tablets, and teriflunomide are present, but unlike European labelling, there is no support for use of ocrelizumab, natalizumab, alemtuzumab, or DMF. INF*β* can be used with caution according to risk/benefit assessment in the USA, while US regulators consider that data on glatiramer acetate are insufficient to reach a conclusion.

### 3.2. Practical Considerations regarding DMD-Based Therapy for Women Who Are or Plan to Become Pregnant

#### 3.2.1. Continuously Administered DMDs

Consideration of a female patient's plans for starting a family is important when prescribing a DMD. In the ideal situation, the patient will start on (and respond to) a DMD, make the necessary changes to the treatment regimen when she decides to become pregnant, wait until the original DMD has been cleared from the system if necessary, promptly become pregnant, and then resume treatment. In practice, needing to stop changing, a DMD is disruptive for the patient and risks a resumption of disease activity. There is also a real risk of an unplanned pregnancy being exposed to a DMD before it is discovered.

Table 1 summarises instructions from European labelling on the administration of DMDs with respect to family planning. Treatment with INF*β* (and possibly GA) can be continued into the pregnancy and so represent a rational choice for therapy for patients with lower disease activity who may wish to become pregnant. Ocrelizumab has a long recommended washout period (12 months) before a patient should become pregnant. The recommended washout period for fingolimod is shorter (2 months), but withdrawal of this DMD risks a rebound reactivation of MS disease activity [20, 21]. Siponimod, which has a similar cellular mechanism of action to fingolimod [22], has a much shorter half-life and a washout period of only 10 days. The current European indication for this agent is secondary progressive MS with active disease, rather than RRMS, however.

If a patient is considered at risk of recurrence of disease activity after withdrawal of fingolimod or siponimod, we would recommend either bridging with INF*β* until treatment can be resumed postpartum or switching to natalizumab if the patient has high MS activity requiring a high-efficacy DMD. We also recommend continuing existing natalizumab therapy until about week 30 of the pregnancy, depending on the needs of the individual patient. Use of natalizumab later in the pregnancy can result in mild-to-moderate anaemia and thrombocytopenia in the neonate [23].

#### 3.2.2. Immune Reconstitution Therapies

Immune reconstitution therapy (IRT) has emerged in recent years as an alternative to continuous application of DMD treatment [24]. Cladribine tablets (oral administration) and alemtuzumab (given by infusion) are the two agents currently available that are believed to act as IRTs, and which are not administered continuously. Both are considered high-efficacy DMDs [19, 25, 26].

**Figure 1:**
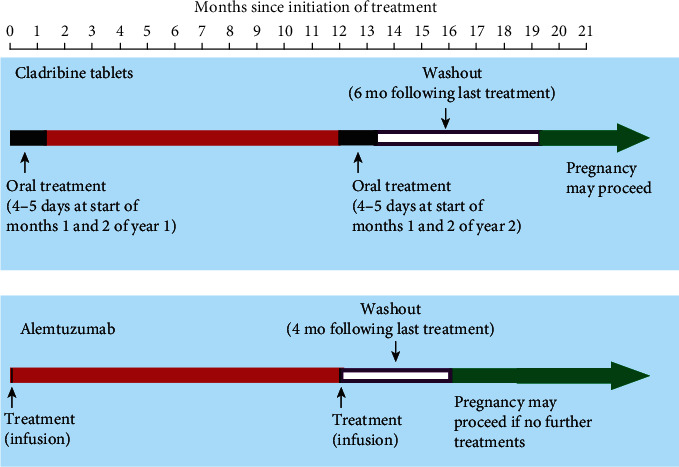
Schematic representation of the timings of treatment with disease-modifying drugs for MS that are hypothesised to act as immune reconstitution inhibitors, with regard to planning a pregnancy. Timings refer to the second year of a two-year course of treatment. If a patient becomes pregnant after only one course of treatment, the second course must be delayed until after the pregnancy (see text).

Clinical findings with both agents demonstrate a potential for a prolonged disease-free period following the 2-year treatment course, in which evidently long after effects on lymphocytes have reversed [24–26]. A patient with higher MS disease activity requiring a high-efficacy DMD, but who does not want to take a DMD during her pregnancy, may be willing to consider a trade off, where she waits until after the IRT treatment course and its recommended washout period (4 months for alemtuzumab, 6 months for cladribine tablets) to become pregnant, in return for a good possibility of conducting her pregnancy uncomplicated by either relapses or treatment with a DMD. This waiting time would be ~20 months for cladribine tablets or ~16 months for alemtuzumab (Figure 1).

### 3.3. Current State of the Art on the Teratogenicity of DMDs

Research into the clinical pharmacology and safety of DMDs continues, and drug labels necessarily do not always reflect the current state of knowledge.  Table 2 provides a brief overview of current evidence relating to the safety of DMDs during pregnancy [27–44]. The large number of pregnancies exposed to INF*β* underpins the recent relaxation of its European label with respect to use in pregnancy. The reproductive safety of GA is also supported by a substantial evidence base, although its label has not been altered to reflect this at the time of writing. Other DMDs are not yet supported by sufficient evidence to support an indication for use in pregnancy, although most physicians will be prepared to use natalizumab in pregnancy for a patient with high disease activity who requires a more efficacious drug than INF*β*. Data on unplanned pregnancies in patients exposed to cladribine tablets are also reassuring, although this DMD retains a formal contraindication for use in pregnancy. Further studies, especially from pregnancy registries, will continue to inform the appropriate therapeutic use of DMDs in pregnant women with RRMS.

Teriflunomide is the active metabolite of leflunomide, which is used in the management of rheumatoid arthritis. Accordingly, the section of Table 2 that deals with teriflunomide also includes data on leflunomide-exposed pregnancies, for completeness. Few data are available on the outcomes of pregnancies of women whose partners are taking DMDs for RRMS. Two studies reported no adverse pregnancy outcomes from a total of 254 pregnancies of women with partners taking teriflunomide or leflunomide [42, 45].

In general, these findings are reassuring, with most reports (other than the EMA's analysis of data on fingolimod) not suggesting the presence of marked teratogenic effects of these DMD. This information may be especially useful for counselling and advising a patient who becomes pregnant unexpectedly while taking a DMD for RRMS.

## 4. Disease-Modifying Drugs and Breastfeeding

INF*β* is the only DMD with an unequivocal indication for use during breastfeeding, in its European label. Excretion of injected INF*β* into breast milk is minimal: one study showed that the dose of INF*β*_1a_ that an infant would receive via breast milk would equate to 0.0006% of the dose received by the mother [46]. Moreover, INF*β* is not active when given orally, as shown by a randomised study involving six months of oral administration of INF*β*, at half the maximal weekly dose given subcutaneously for the management of RRMS [47]. There was no evidence of a systemic effect, relative to placebo. These data are consistent with an absence of clinically significant exposure of the neonate to INF*β* during breastfeeding.

Natalizumab is excreted into breast milk, providing doses to the infant that may have functional effects [48, 49]. Women taking this DMD postpartum should not breastfeed. Alemtuzumab, DMF, and GA may be used during breastfeeding subject to a risk:benefit evaluation in Europe. Cladribine tablets, fingolimod (and siponimod), natalizumab (see above), ocrelizumab, and teriflunomide have formal contraindications to use in breastfeeding in Europe. US labelling permits the use of most DMDs during breastfeeding, again according to an evaluation of benefits and risks; only cladribine tablets and teriflunomide have an outright contraindication in this setting. The rapid elimination of cladribine from the system permits breastfeeding as soon as 1 week after cessation of treatment with this agent, according to its European labelling.

## 5. Expert Opinion on Impact of Issues Related to Family Planning on Prescribing a DMD for a Woman of Childbearing Age

The expert authors of this article undertook an exercise to rank the importance of a series of seven preselected issues (see Table 3) relating to the therapeutic use of DMDs in women of childbearing age. First, authors allotted a score of 1 (low impact) to 5 (high impact) as to their importance of these issues as potential drivers of a prescribing decision; the average score for each allowed ranking in terms of importance, as shown below, in descending order of importance. The presence of a clinical indication for use during pregnancy was the highest-rated factor, following known low risk of teratogenicity, and a short washout period in case of a need to switch treatment in the event of a pregnancy. The existence of positive clinical data for treatment-exposed pregnancies was rated the next highest, and the relatively low ranking of this issue reflected the general lack of data available for most DMDs. Provision of a time window of controlled disease to permit the course of a pregnancy was rated the next highest. Finally, issues relating to lactation (therapeutic indication and existence of positive data) were the rated least important.

The impact of issues relating to potential pregnancy and lactation were considered separately for individual DMDs, according to the patient's level of MS disease activity. Consideration of possible future pregnancy would drive prescription of INF*β*, and to a slightly lesser extent glatiramer acetate, more strongly than DMF or teriflunomide for a patient with mild-to-moderate disease activity. This was consistent with the greater availability of clinical data for INF*β* and glatiramer acetate in this setting. Positive evidence for natalizumab in pregnancy, and its lack of a formal contraindication in pregnancy in Europe, drove higher ratings of this agent for women of childbearing potential who had high disease activity. The potential of IRT to provide a disease- and treatment-free window of opportunity to pursue a pregnancy drove a high rating for cladribine tablets associated with this issue, although general restrictions on the use of alemtuzumab due to potential safety concerns were noted. Formal contraindications during pregnancy (and need to withdraw treatment should pregnancy occur), and long washout periods, impacted negatively on prescribing of ocrelizumab and fingolimod.

## 6. Conclusions

A diagnosis of RRMS has been perceived as a barrier to achieving a fulfilling family life for too long. Several studies based on registries, described above, show that a substantial minority of women undergo elective termination of unplanned pregnancies that have been exposed to DMDs taken for RRMS [27–44]. Perceptions of the barriers to having a family of women with MS appear to be reducing: data from the USA showed that the proportion of women with MS who are pregnant increased steadily between 2006 and 2014, in contrast to a decreasing proportion of women without MS who were pregnant over the same period [50]. This is a welcome development, which must be continued. It is important that physicians continue to reassure and support women with MS (and their partners) in feeling confident that they can raise a family, including providing support and reassurance for breastfeeding, where this is possible.

It remains important to prevent relapses as far as possible during pregnancy, and the increasing choice of DMDs has provided more flexibility here. Practical therapeutic options for treatment up to and including pregnancy are now available for women with active MS but lower disease activity who require active treatment. For example, INF*β* now has a formal therapeutic indication for such patients, and in our experience, this development has helped to persuade patients of the benefits of maintaining treatment during pregnancy, who would otherwise be reluctant to continue treatment. GA represents a further possible option for these patients. Women with higher levels of disease activity requiring maintained treatment with a high-efficacy DMD could in principle be treated with natalizumab (to 30 weeks), or possibly with an IRT for women who are prepared to delay their pregnancy until the treatment course and associated washout period have been completed.

## Figures and Tables

**Table 1:** Practical considerations relating to the use of disease-modifying drugs before and during pregnancy, according to European labelling.

DMD	Potential for use before and during pregnancy
Alemtuzumab^a,b^	Maintain contraception for 4 months after the end of the second-year treatment course^c^.
Cladribine tablets^a^	Maintain contraception for 6 months after the end of the second-year treatment course.
Dimethyl fumarate	No recommendation given on washout period.
Fingolimod^a^	Maintain contraception for 2 months after the last dose. Withdraw immediately if pregnancy is discovered.
Glatiramer acetate	No recommendation given on washout period.
Interferon beta	Can be continued into pregnancy if clinically needed.
Natalizumab^a^	No recommendation given on washout period.
Ocrelizumab^a^	Maintain contraception for 12 months after the end of the second-year treatment course.
Siponimod^a^	Maintain contraception for 10 days after the last dose. Withdraw immediately if pregnancy is discovered.
Teriflunomide	Women should not become pregnant until plasma levels of teriflunomide are <0.02 mg/L (average 11 days if the accelerated elimination procedure is used, 8–24 months if not). Withdraw immediately if pregnancy is discovered (use accelerated elimination procedure).

^a^High-efficacy DMD (see reference [19]). ^b^Can be used in pregnancy if clinically justified (benefits to mother outweigh risks to the foetus). Collated from European Summaries of product Characteristics (available at http://www.medicines.org.uk/, accessed October 2020). See text for differences from US Prescribing Information.

**Table 2:** Summary of evidence relating to the foetal safety of DMDs used for relapsing-remitting multiple sclerosis.

DMD	Overview of evidence relating to safety during pregnancy
Alemtuzumab^a^	(i) No apparent increase in the frequency of spontaneous abortions between women who had received alemtuzumab and women in the general population (2017, based on 248 pregnancies) [27].

Cladribine tablets^a^	(i) Similar proportions of live births and spontaneous abortion in women who received cladribine tablets or placebo during the clinical development of this DMD (2017, based on 64 pregnancies) [28].

Dimethyl fumarate	(i) No signal for adverse pregnancy outcomes in 63 women in clinical trials and 125 pregnancies described postmarketing (2015) [29]. (ii) International Registry data (194 pregnancies) showed unremarkable rates of pregnancy loss and birth defects (2019) [30].

Fingolimod/siponimod^a^	(i) Prospective Multinational Gilenya® Pregnancy Exposure Registry found a rate of birth defects consistent with the range found in the general population (based on 1,586 pregnancies, 2019) [31]. (ii) A review by the EMA found a 2-fold increase in the rate of birth malformations (2019) [32].

Glatiramer acetate (GA)	(i) Registry data suggest no teratogenic effect (based on 246 pregnancies, 151 with exposure in the 1st trimester, 3 to the 3rd trimester, 95 unexposed controls, 2016) [33]. (ii) Comparison of a database including 5,042 pregnancies exposed to GA with control databases including 29% of the European births (>1.7 million/year) and >50,000 births in the USA showed no excess birth defects or other adverse pregnancy outcomes (2018) [34].

Interferon beta (INF*β*)	(i) 2,148 exposed and 2,025 unexposed pregnancies from the German Multiple Sclerosis and Pregnancy Registry (2016), the Merck Serono Global Drug Safety Database (2011), and a Nordic Pregnancy Registry (2018) showed no excess risk to the foetus resulting from exposure o INF*β* (live births, spontaneous abortions, congenital abnormalities, and birth length/weight) relative to the general population [35–37].

Natalizumab^a^	(i) Registry data included 101 women with RRMS foetal exposure to natalizumab, 78 women with RRMS and pregnancy unexposed to natalizumab, and 97 control; non-MS pregnancies demonstrated no significant differences for major malformations, low birth weight (<2500 g), or premature birth (2015) [38]. (ii) Observational data suggested odds ratio of 3.9 for spontaneous abortion with natalizumab vs. INF*β* or no treatment (*p*<0.001); the frequency of spontaneous abortion (17.4%) and of major congenital abnormalities (3.7%) was within estimates for the local general population (92 exposed pregnancies 2018) [39]. (iii) No excess risk of miscarriages or birth defects global Tysabri Pregnancy Exposure Registry (376 pregnancies, 2016) [40].

Ocrelizumab^a^	(i) No signal for increased rates of spontaneous abortion in for 267 pregnancies (118 with documented foetal exposure). No foetal abnormalities were reported for 26 live births from these women (2019) [41].

Teriflunomide	(i) Spontaneous abortion rate of 18.6% from 70 pregnancies with known exposure to teriflunomide; this was described as within the expected range for the general population (2019) [42]. (ii) Spontaneous abortion rate of 21% from 431 exposed pregnancies, and 4 birth defects (these were considered consistent with the rate in the general population (2019) [43]. (iii) 587 pregnancies exposed to leflunomide (for arthritis) did not suggest teratogenic potential (7% birth defects; 2019) [44].

Dates are years of publication or presentation at an international meeting. EMA: European Medicines Agency. RRMS: relapsing-remitting multiple sclerosis. ^a^Usually considered a high-efficacy disease-modifying drug (DMD) for the management of RRMS (see reference [19]).

**Table 3:** Authors' rating of issues relevant to the therapeutic use of a DMD in women of childbearing potential.

Issue	Average rating
Existence of a therapeutic indication or use during pregnancy	4.5
Known low risk of teratogenicity	4.3
Short washout period if withdrawn	4.1
Positive data from DMD-exposed pregnancy	3.7
Provision of a sufficient period of controlled disease to complete a pregnancy	3.5
Existence of a therapeutic indication or use during lactation	3.3
Positive data from breastfeeding women taking the DMD	2.9

Experts allotted a score of 1 (low) to 5 (high) reflecting the importance of each issue as a driver of prescribing decisions for a woman of childbearing potential, and average scores for each issue are shown here.
